# Serum concentration of several trace metals and physical training

**DOI:** 10.1186/s12970-017-0178-7

**Published:** 2017-06-12

**Authors:** Marcos Maynar, Francisco Llerena, Francisco J. Grijota, Javier Alves, María C. Robles, Ignacio Bartolomé, Diego Muñoz

**Affiliations:** 1 Sport Sciences Faculty, University of Extremadura, Avenida de la Universidad s/n, 10003 Cáceres, Spain; 2 School of Medicine, University of Extremadura, Avda. de Elvas s/n, 06071 Badajoz, Spain; 3 Education Faculty, University of Salamanca, C/Henry Collet, 52–70, 37007 Salamanca, Spain

**Keywords:** Minerals, Blood, Aerobic, Anaerobic, Mass spectrometry

## Abstract

**Background:**

The aim of this study was to observe the concentrations of trace metals boron, lithium, rubidium, antimony, tin and strontium in the serum of athletes from different modalities and sedentary subjects and the possible influence that different energy sports training modalities can have on their concentration.

**Methods:**

Eighty professional athletes and 31 sedentary males participated in the present survey. All of them were living in Cáceres (Spain). Serum boron, lithium, rubidium, antimony, tin and strontium analysis was performed by Inductively Coupled Plasma Mass Spectrometry (ICP-MS).

**Results:**

The results show higher concentrations in athletes on tin (*p* < 0.01), rubidium and antimony (*p* < 0.001) than the control group. In the case of tin, this item had the highest concentrations only in aerobic sports modalities. Regarding rubidium and antimony, the highest concentrations are found in athletes with lower oxygen consumption (aerobic-anaerobic) (*p* < 0.001), followed by anaerobic group (*p* < 0.001).

**Conclusion:**

Our research shows that, probably due to increased water and air intake, especially, trace elements rubidium, antimony and tin reveal major differences in serum concentration of athletes in relation to sedentary subjects. On the other hand, physical training does not change the serum concentration of Boron, Lithium and strontium.

## Background

Currently, the roles of main essential metals (selenium, zinc, copper) in the body and their relationship with human cellular functions and physical activity are known. However, little information about trace metals boron (B), lithium (Li), rubidium (Rb), antimony (Sb), tin (Sn) and strontium (Sr) has been established and no study discusses the effects that physical training can have on them. Nevertheless, these elements are present in the human body in different concentrations and we do not understand their possible roll in body functions.

Contents of B in human tissues vary between <0,2 and <0,5 mg kg^−1^, being the highest in the kidneys and liver and the lowest in skin [1]. Its concentrations in soft tissues of humans range from 0,06 to 0,6 mg kg^−1^, in the brain and kidneys, respectively. The average content in tissues of the “reference man” is 0,3 mg kg^−1^ [2]. Metabolism of vitamin D and estrogens, as measured by plasma metabolites, macromineral (especially calcium) metabolism, and immune function, have been proposed as related to a function for B in humans [3–5].

Li occurs in human tissues within the very narrow range of <0,02 to 0,08 mg kg^−1^, being the most concentrated in skin [1]. The average Li content is estimated for total soft tissues of humans as 0,006 mg kg^−1^ [2]. Clinical studies suggest that Li causes desensitization of adrenergic receptor α-2, showing that its primary site of action focuses on mechanisms of intracellular communication [6]. Li exerts a direct inhibition of adenylate cyclise, alters processes mediated by cyclic adenosine mono-phosphate (AMP) and intracellular ionic transport mechanisms [7].

Rb is present in all tissues of humans within the range of 8 to 30 mg kg^−1^, with the lowest value for skin and the highest for the liver [1]. Total average soft tissue content of Rb in “reference man” has been calculated at 9,7 mg kg^−1^ [2]. There is some evidence that Rb is involved in brain functions, but specific roles have not yet been identified [8].

Sb occurs in human tissue in the range of 5 to 10 μg kg^−1^, being the lowest in the heart and the highest in muscles, its average for total soft tissue is estimated to be 9,4 μg kg^−1^ and its content in “reference man” is calculated at 30 μg kg^−1^ [2]. Sb is a cumulative poison [9]. Average contents of Sb in food products are between 0,2 and 1,1 μg kg^−1^.

Human tissues contain Sn at the range of <0,2–0,85 mg kg^−1^, being the lowest in the brain and the highest in the liver and kidneys [1]. According to Li [2], total soft tissues of humans contain Sn at 0,1 mg kg^−1^, and its content of all tissues of a “reference man” averages at 0,24 mg kg^−1^. Increased Sn concentration in food may cause acute gastric irrigation, impaired reproductivity, and bone strength failure [10].

Sr occurs in all human tissues in the range of 0,09 to 0,24 mg kg^−1^. Its highest concentration is in the kidneys and lowest in the brain [1]. It is accumulated mainly in bones. Biochemical functions of Sr are not well known, but as reported by D’Haese et al. [11], its small quantities are needed for proper processes of the calcification of bones and teeth.

Considering the above, the objective of this study is to observe the concentrations of these trace elements in the serum of athletes from different modalities and sedentary subjects and the possible influence that different energy sports training modalities can have on their concentration.

## Methods

### Participants

This research was carried out under the Helsinki Declaration ethic guidelines, updated at the World Medical Assembly in Seoul in 2008, for research with human subjects. All the participants were informed about the purpose of the study and gave their voluntary signed informed consent.

Eighty professional athletes and 31 sedentary males participated in the present survey. All of them were living in Cáceres (Spain). The athletes had trained regularly for the two previous years. They all completed a nutritional questionnaire about their eating habits to ensure they were not taking any vitamins, minerals or other supplementation and to guarantee they all had a similar diet.

The athletes were classified into four groups: athletes group (athletes; *n* = 80) of all the modalities (aerobic + anaerobic + aerobic-anaerobic) with an average age of 20,3 ± 3,2 years; aerobic athletes group (Aerobic: long distance runners; *n* = 28) average aged of 21,5 ± 4,3 years; anaerobic athletes group (Anaerobic: judo and speed athletes; *n* = 24) with an average age of 20,1 ± 2,5 years; and aero-anaerobic athletes group (Aerobic-anaerobic: professional football players; 28 individuals) with and average age of 22,2 ± 4,3. Sedentary students, with an average age of 21,7 ± 3,5 years, formed the control group.

Anthropometric and cardiorespiratory characteristics of all participants are shown in Tables 1 and 2. None of them smoked or consumed supplements of the metals studied.

**Table 1:** Characteristics of the groups in the various sporting metabolic modalities and control group

	Control (*n* = 31)	Sportsm (*n* = 80)	Aerobic (*n* = 28)	Anaerobic (*n* = 24)	Aerobic-anaerobic (*n* = 28)
Height (m)	1,76 ± 0,057	1,76 ± 0,07	1,77 ± 0,05	1,73 ± 0,07	1,80 ± 0,05
Weight (kg)	78,21 ± 12,19	65,31 ± 7,55***	64,95 ± 7,10***	64,91 ± 8,46***	73,78 ± 6,12*†††■■■
Σ4 skinfolds (mm)	52,02 ± 23,77	35,12 ± 9,29***	32,56 ± 8,75***	33,66 ± 9,87***	38,25 ± 10,06**†
Σ6 skinfolds (mm)	88,82 ± 34,5	45,85 ± 16,69***	49,69 ± 14,84***	56,32 ± 16,65**††	59,49 ± 17,10**†††
Fat weight (kg)	9,60 ± 3,32	5,94 ± 1,27***	5,54 ± 1,05***	6,16 ± 1,45**††	7,03 ± 1,05***†††■
Muscle weight (kg)	36,60 ± 4,18	31,51 ± 3,72***	31,96 ± 3,68***	30,67 ± 4,1***	36,14 ± 3,33†††■■■
Bone weight (kg)	11,77 ± 2,02	12,26 ± 1,63	12,03 ± 1,27	12,43 ± 2,15	12,83 ± 0,92*††
Fat percentage (%)	12,28 ± 2,87	9,01 ± 1,23***	8,45 ± 1,03***	9,39 ± 1,27***††	9,51 ± 1,03***†††
Muscle percentage (%)	48,21 ± 3,31	48,13 ± 2,01	48,93 ± 1,58	47,32 ± 2,68††	48,92 ± 1,28■■
Bone percentage (%)	15,37 ± 1,63	18,76 ± 1,87***	18,5 ± 1,63**	19,19 ± 2,46***	17,39 ± 0,9***††■■

Anova and post oc Bonferoni test. (*p < 0.05; **p < 0.01; ***p < 0.001) differences between control group vs athletes, aerobic, anaerobic and aerobic-anaerobic groups; († *p* < 0.05, †† *p* < 0.01, ††† *p* < 0.001) differences between aerobic vs anaerobic group and aerobic-anaerobic group; (■ *p* < 0.05; ■■ *p* < 0.01; ■■■ *p* < 0.001) differences between anaerobic vs aerobic-anaerobic group

**Table 2:** Ergospirometric parameters of the groups in the various sporting metabolic modalities and control group

	Control (*n* = 31)	Sportsm (*n* = 80)	Aerobic (*n* = 28)	Anaerobic (*n* = 24)	Aerobic-anaerobic (*n* = 28)
VO2Máx (mL/min/kg)	43,93 ± 7,28	64,08 ± 5,27***	66,17 ± 8,362***	61,23 ± 3,004***†	59,85 ± 4,539**††■
Resting HR (b/min)	72,58 ± 7,38	59,75 ± 9,66*	54,90 ± 10,85**	62,85 ± 7,57*††	57,21 ± 10,51*■
Max HR (b/min)	187,11 ± 5,38	196,41 ± 6,64***	194,03 ± 7,25**	198,65 ± 4,93***†	188,79 ± 11,83†■■

Anova and post oc Bonferoni test. (*p < 0.05; **p < 0.01; ***p < 0.001) differences between control group vs athletes, aerobic, anaerobic and aerobic-anaerobic groups; († *p* < 0.05; †† *p* < 0.01) differences between aerobic vs anaerobic group and aerobic-anaerobic group; (■ *p* < 0.05; ■■ *p* < 0.01) differences between anaerobic vs aerobic-anaerobic group

### Anthropometric measurements

The participants’ morphological characteristics were measured in the afternoon and always at the same time (09:00 A.M). Body height was measured to the nearest 0,1 cm using a wallmounted stadiometer (Seca 220), and body weight was measured to the nearest 0,01 kg using calibrated electronic digital scales, (Seca 769) in barefoot conditions. Body mass index was calculated by dividing the weight (in kg) by the height (in m^2^). Body fat content was estimated from the sum of 4 skinfolds (abdominal, suprailiac, tricipital and subescapularis) and from the sum of 6 skinfold (∑4+ thigh and calf skinfolds). The skinfolds thicknesses were measured with a Harpenden calliper. Body composition was calculated as shown in the kinanthropometry Spanish group [12]. The same operator, skilled in kinanthropometry techniques, made all measurements.

### Exercise test

To measure the different fitness levels of the athlete and control groups, a maximal progressive exercise test was performed. The protocol of the test consisted in running on a treadmill (Powerjoc. Uk), until exhaustion at a starting speed of 10 km/h, which increased 1 km/h every 400 m, with a stable slope of 1%. To perform the test, a Polar pulsometer (Polar. Norway) and an ergospirometer system equipped with a gas analyzer (Metamax. Cortex Biophysik. Gmbh. Germany) were used. Ergospirometers parameter’s determinates were VO_2_ max, VCO_2_ max, VE max, resting heart rate and others.

### Sample collection

At nine o’clock in the morning, after weighing the participants, five millilitres of antecubital venous blood were drawn from each participant using a plastic syringe with a stainless steel needle. The blood sample was collected in a metal-free polypropylene tube (previously washed with diluted nitric acid). Then, the blood sample was centrifuged at 3000 rpm for 15 min at room temperature (23 ± 1 °C) to separate the serum. The serum was aliquoted into an Eppendorf tube (previously washed with diluted nitric acid) and was left to stand at −80 °C until further analysis.

### Elements determination

Serum B, Li, Rb, Sb, Sn and Sr analysis was performed by Inductively Coupled Plasma Mass Spectrometry (ICP-MS) in accordance with Sarmiento-González et al. [13]. Decomposition of the organic matrix was performed by heating it at 90 °C for 10 h after the addition of 0,8 mL HNO_3_ and 0,4 mL H_2_O_2_ to 2 mL of serum. The samples were then dried at 200 °C on a hot plate. Sample reconstitution was carried out by adding 0,5 mL of nitric acid, 10 μL of Indium (In) (10 mg/L) as internal standard and suprapure water to 10 mL. Digested solutions were assayed by ICP-MS NexION model 300D (PerkinElmer, Inc., Shelton, CT, USA). Three replicates were analysed per sample. Quantification was performed by In as internal standard. The values of the standard materials of each element (10 μg/L) measured for quality control were in good agreement with intra and inter-assay variation coefficients of less than 5%.

### Statistical evaluations

In the study, serum concentrations of toxic elements in the control group and in all the athlete participants (*n* = 80) were compared to investigate if there were any changes. After this, the control group was compared with athletes of different modalities to determine if there were different influences according to the modality practiced.

Statistical analyses were performed with the *IBM SPSS Statistic* software version 19 for *Windows*. The results are expressed as *x* ± *s*, where *x* are the mean values and *s* the standard deviation. The normal distribution of the variables was assessed using the Shapiro-Wilks test. Data were analyzed for significant differences between mineral concentrations in serum samples of the four groups using one-way Anova followed by Bonferroni post hoc test. A *p* < 0.05 was considered statistically significant.

## Results

### Anthropometric characteristics

In this research, well-characterized groups were studied. In Table 1, data obtained of the anthropometric characteristics from the five groups are presented. It can be observed that athletes had a total body weight and fat values (**Σ** of skinfolds) significantly lower (*p* < 0.001) than the control group. Aerobic and anaerobic athletes showed lower (*p* < 0.001) body weight, followed by aerobic-anaerobic athletes (*p* < 0.05). In relation to fat, aerobic athletes have the least (*p* < 0.001) values followed by anaerobic (*p* < 0.001) and aerobic-anaerobic (*p* < 0.01).

### Ergospirometric parameters

VO_2_ max was significantly higher (*p* < 0.001) in athletes compared to the sedentary group. Resting heart rate was lower (*p* < 0.01) in athletes with respect to the control group. Maximum heart rate was higher (*p* < 0.01) in athletes with respect to the control group.

In relation to the oxygen update, controlled to assess the cardiovascular fitness of the study subjects, it is the aerobic athletes who have a higher (*p* < 0.001) value followed by anaerobic (*p* < 0.001) and aerobic-anaerobic (*p* < 0.01) with respect to the control group. As for resting heart rate, it was lower (*p* < 0.01) in aerobic followed by aerobic-anaerobic (*p* < 0.05) and anaerobic athletes (*p* < 0.05). For maximum heart rate, it was lower in aerobic-anaerobic followed by aerobic (*p* < 0.05) and anaerobic athletes (*p* < 0.01).

### Serum concentrations of B, Li, Rb, Sb, Sn and Sr

Table 3 shows the data obtained in the study for the elements B, Li, Rb, Sn, Sb, Sr and V. When compare the athlete group with the control group found that much higher concentrations were observed in the group of athletes on Sn (*p* < 0.01) and Rb and Sb (*p* < 0.001) than in the control group. In the case of Sn, this item had the highest concentrations only in aerobic sports modalities. Regarding Rb and Sb, the highest concentrations are found in athletes with lower oxygen consumption (anaerobic) (*p* < 0.001), followed by aerobic-anaerobic (*p* < 0.001). However, aerobic athlete specialties are similar to the control group and had significantly lower concentrations (*p* < 0.001) than other groups. Similar results were found for Sb.

**Table 3:** Serum concentrations of B, Li, Rb, Sb, Sn, Sr and V of the control group and athletes from different modalities

	Control (*n* = 31)	Sportsm (*n* = 80)	Aerobic (*n* = 28)	Anaerobic (*n* = 24)	Aerobic-anaerobic (*n* = 28)
B (μg/L)	5,002 ± 2,116	5,731 ± 4,117	6036 ± 4541	5,267 ± 2,655	5,106 ± 4,234
Li (μg/L)	1,466 ± 1,681	1,448 ± 0,634	1343 ± 0,854	1,365 ± 0,465	1,619 ± 0,535
Rb (μg/L)	147,01 ± 24,99	211,87 ± 53,59**	163,90 ± 55,22	254,42 ± 30,48**†††	219,38 ± 20,03**†††■■
Sb (μg/L)	0,999 ± 0,235	3,440 ± 2,148**	1,487 ± 1,982	4,945 ± 0,876**†††	4,143 ± 1,229**†††■
Sn (μg/L)	0,214 ± 0,426	0,833 ± 1,410*	1,431 ± 1,465**	0,128 ± 0,114†††	0,457 ± 1,443††
Sr (μg/L)	30,67 ± 7,483	32,17 ± 9,935	29,65 ± 10,19	34,86 ± 10,03†	32,13 ± 8,546

Anova and post oc Bonferoni test. (*p < 0.01; **p < 0.001) differences between control group vs athletes, aerobic, anaerobic and aerobic-anaerobic groups; († *p* < 0.05; †† *p* < 0.05; ††† *p* < 0.001) differences between aerobic vs anaerobic group and aerobic-anaerobic group; (■ *p* < 0.01; ■■ *p* < 0.001) differences between anaerobic vs aerobic-anaerobic group

## Discussion

Physical activity leads to many metabolic changes in the body and therefore regular intense exercise training may increase mineral requirements, either by increasing degradation rates or by decreasing losses from the body. Also, continued physical activity can lead to an increase in the total intake of water and air, which can lead to trace elements, such as those studied, entering the organism through these paths, which may cause alterations in the functioning of the bodies of athletes.

Our results show that performing continuous physical activity can cause changes in serum concentrations of some of these elements.

Thus, we can find that the concentration of B in humans is 0–0,13 μg/L [2]. In our work we found values within the normal range and showed no significant differences when comparing controls with athletes.

Lu et al. [14] found Li plasma concentrations of 1,90 ± 0,04 μg/L that remain similar to those of our study, and showed no significant differences between the study groups.

Rb has an intracellular distribution similar to that of potassium (K) and may act as an antagonist and sometimes substitute of it. Rb competes with K in membrane transport processes and is displaced in various cell types, including muscle cells and erythrocytes [10]. It inhibits some potassium-dependent enzymes, but can activate others [15]. The concentrations found in serum of Rb are 190 ± 40 μg/L [14]; similar values found in our subjects. Rb was found in higher concentrations in athletes in relation to controls but only in athletes who used the anaerobic system, not in aerobic athletes. This could be due to the fact that higher amounts of rubidium in the body are in muscle tissue, which has a special affinity for alkali metals such as Rb and K [1]. This is perhaps the reason why the highest levels in our study are in athletes with more muscle volume and to a lesser extent when less muscle volume is present. Rb is associated with the K cycle in organisms but does not substitute for it [10]. So Rb can help K in the extreme situation that is intense exercise.

Exposure of humans to Sb can occur through breathing, drinking water and food containing it, but also by skin contact with soil, water and other substances that have it [16]. In our study, the serum concentration of Sb was between 0 and 2 μg/L, which was indicated by Repetto and Repetto [17]. Sb had a similar occurrence in the case of Rb distribution among athletes. Sb was in higher concentrations in athletes in relation to controls but only in athletes who used the anaerobic system, not in aerobic athletes. This could be due to the fact that higher amounts of Sb in the body are in muscle tissue [2] and most anaerobic athletes had more volume of muscle than aerobic athletes.

Humans are exposed to Sn from ingestion, inhalation, and dermal adsorption. Canned foods, especially fruits and vegetable products, are considered to be the main source of Sn in the diet. Some canned fruits and juices may contain Sn at the range of 141 to 2000 mg kg^−1^. It is, perhaps, the increased water intake and increased mobilization of air for aerobic athletes which is the cause of higher serum concentrations in these athletes and not in the other exercise modalities. We found no data in the literature that can clarify this situation that occurs in our athletes.

Sr is found in humans at a concentration of 25 ± 8 μg/L [14], similar to that found in our study. But no significant differences between groups were found.

At the present, these elements are not of concern to human health, but it would be interesting to do more studies addressing their possible relationship to athletic performance.

## Conclusions

Our research shows that, probably due to increased water and air intake, especially, trace elements Rb, Sn and Sb reveal major differences in serum concentration of athletes in relation to sedentary subjects and serum concentrations have relation with the type of physical training. On the other hand, physical training does not change the serum concentration of B, Li and Sr.

## References

[1] Jørgensen SE (2000). Principles of pollution abatement. Pollution abatement for the 21st century.

[2] Li YH (2000). A compendium of geochemistry: from solar nebula to the human brain.

[3] Nielsen FH (1998). The justification for providing dietary guidance for the nutritional intake of boron. Biol Trace Elem Res.

[4] Nielsen FH, Penland JG (1999). Boron supplementation of peri-menopausal women affects boron metabolism and indicies associated with macromineral metabolism, hormonal status and immune function. J Trace Elem Exp Med.

[5] Samman S, Naghii MR, Lyons Wall PM, Verus AP (1998). The nutritional and metabolic effects of boron in humans and animals. Biol Trace Elem Res.

[6] Risby ED, Hsiao JK, Manji HK, Bitran J, Moses F, Zhou DF, et al. The mechanisms of action of lithium. II. Effects on adenylate cyclase activity and beta-adrenergic receptor binding in normal subjects. Arch Gen Psychiatry. 1991;48:513–24. 10.1001/archpsyc.1991.01810300025004 1645514

[7] Lydiard RB, Gelenberg AJ (1982). Hazards and adverse effects of lithium. Annu Rev Med.

[8] Hameed A, Vohra SB (1990). Role of elements in pathogenesis and therapy of some selected diseases, new horizon of health aspects of element.

[9] Shotyk W, MacKenzie A, Norton S (2004). Archives of environmental contamination. J Environ Monitor.

[10] Kabata-Pendias A, Mukherjee AB (2007). Trace elements from soil to human.

[11] D’Haese PC, Verbeckmoes SC, Schrooten I, De Broe ME (2002). The effects of strontium on bone formation. Epidemiological experimental and cell biological aspects. Macro and trace elements.

[12] Porta J, Galiano D, Tejedo A, González JM (1993). Valoración de la composición corporal. Utopías y realidades. Manual de Cineantropometría.

[13] Sarmiento-González A, Marchante-Gayón JM, Tejerina-Lobo JM, Paz-Jiménez J, Sanz-Medel A (2005). ICP-MS multielemental determination of metals potentially released from dental implants and articular prostheses in human biological fluids. Anal Bioanal Chem.

[14] Lu Y, Ahmed S, Harari F, Vahter M (2015). Impact of Ficoll density gradient centrifugation on major and trace element concentrations in erythrocytes and blood plasma. J Trace Elem Med Biol.

[15] Russin TZ, Laine RA, Turco SJ (1981). Cell-free biosynthesis of erythroglycan in a microsomal fraction from K-562 cells. Biochem J.

[16] Biégo GH, Joyeux M, Hartemann P (1999). (1999) determination of dietary tin intake in an adult French citizen. Arch Environ Contam Toxicol.

[17] Repetto MR, Repetto M (1999). (1999). Concentrations in human fluids: 101 drugs affecting the digestive system and metabolism. J Toxicol Clin Toxicol.

